# Point of care ultrasound screening for deep vein thrombosis in critically ill COVID-19 patients, an observational study

**DOI:** 10.1186/s12959-021-00272-z

**Published:** 2021-06-02

**Authors:** Sarah Galien, Michael Hultström, Miklós Lipcsey, Karl Stattin, Robert Frithiof, Jacob Rosén, the Uppsala Intensive Care COVID-19 Research Group, Tomas Luther, Sara Bülow Anderberg, Anna Gradin, Sten Rubertsson, Katja Hanslin

**Affiliations:** 1 Department of Surgical Sciences, Anaesthesiology and Intensive Care Medicine, Uppsala University, entrance 78, 1 floor, 751 85 Uppsala, Sweden; 2 Department of Medical Cell Biology, Integrative Physiology, Uppsala University, Uppsala, Sweden; 3 Hedenstierna laboratory, CIRRUS, Department of Surgical Sciences, Anaesthesiology and Intensive Care Medicine, Uppsala University, Uppsala, Sweden

**Keywords:** COVID-19, ICU, Deep vein thrombosis, Screening, Point of care ultrasound

## Abstract

**Background:**

Deep vein thrombosis (DVT) is common in critically ill patients with Coronavirus disease 2019 (COVID-19) and may cause fatal pulmonary embolism (PE) prior to diagnosis due to subtle clinical symptoms. The aim of this study was to explore the feasibility of bedside screening for DVT in critically ill COVID-19 patients performed by physicians with limited experience of venous ultrasound. We further aimed to compare inflammation, coagulation and organ dysfunction in patients with and without venous thromboembolism (VTE).

**Methods:**

This observational study included patients with COVID-19 admitted to the intensive care unit (ICU) of a tertiary hospital in Sweden and screened for DVT with proximal compression ultrasound of the lower extremities between April and July 2020. Screening was performed by ICU residents having received a short online education and one hands-on-session. Pathological screening ultrasound was confirmed by formal ultrasound whereas patients with negative screening underwent formal ultrasound on clinical suspicion. Clinical data, laboratory findings and follow-up were extracted from medical records.

**Results:**

Of 90 eligible patients, 56 were screened by seven ICU residents with no (*n* = 5) or limited (*n* = 2) previous experience of DVT ultrasound who performed a median of 4 (IQR 2–19) examinations. Four (7.1%) patients had pathological screening ultrasound of which three (5.6%) were confirmed by formal ultrasound. None of the 52 patients with negative screening ultrasound were diagnosed with DVT during follow-up. Six patients were diagnosed with PE of which four prior to negative screening and two following negative and positive screening respectively. Patients with VTE (*n* = 8) had higher median peak D-dimer (24.0 (IQR 14.2–50.5) vs. 2.8 (IQR 1.7–7.2) mg/L, *p* = 0.004), mean peak C-reactive protein (363 (SD 80) vs. 285 (SD 108) mg/L, *p* = 0.033) and median peak plasma creatinine (288 (IQR 131–328) vs. 94 (IQR 78–131) μmol/L, *p* = 0.009) compared to patients without VTE (*n* = 48). Five patients (63%) with VTE received continuous renal replacement therapy compared to six patients (13%) without VTE (*p* = 0.005).

**Conclusion:**

ICU residents with no or limited experience could detect DVT with ultrasound in critically ill COVID-19 patients following a short education. VTE was associated with kidney dysfunction and features of hyperinflammation and hypercoagulation.

**Trial registration:**

ClinicalTrials ID: NCT04316884. Registered 20 March 2020.

## Introduction

Hypercoagulation and associated deep vein thrombosis (DVT) is a common and severe consequence of the inflammatory response in critically ill patients with Coronavirus disease 2019 (COVID-19) and has led to implementation of COVID-19 specific thromboprophylaxis regimens [1–7]. Symptoms of DVT in the critically ill are often vague but may cause life-threatening pulmonary embolism (PE) and most patients will be considered high risk for DVT using conventional risk assessment scores, limiting their utility [8]. Routine screening for DVT could therefore be beneficial for early diagnosis of asymptomatic DVT in patients with COVID-19 admitted to intensive care units (ICU) [9].

Complete duplex ultrasound (CDUS) of the entire lower extremity is the recommended imaging technique for evaluation of suspected DVT [10]. CDUS is time-consuming and requires considerable training to perform and interpret and is therefore only performed by designated operators. During the COVID-19 pandemic, screening with CDUS would be limited by availability, expose ultrasound operators to infection and may increase in-hospital contamination [11]. As an alternative to CDUS, two-region compression ultrasound (2-CUS) of the common femoral and popliteal veins only, is accurate for diagnosing DVT in non-COVID-19 patients and can be performed by emergency and critical care physicians [12–14]. Further, 2-CUS point-of-care screening has good agreement with formal ultrasound in critically ill COVID-19 patients [15]. An extended compression ultrasound (ECUS) improves sensitivity by diagnosing thrombi isolated to the superficial femoral vein [16].

DVT screening in COVID-19 patients has been described using ultrasound of the entire lower extremity [17–24], 2-CUS [25] and ECUS [26, 27]. However, in previous studies screening was performed by physicians experienced in DVT studies.

We therefore aimed to investigate the feasibility of bedside screening for DVT in critically ill COVID-19 patients performed by physicians unexperienced in venous ultrasound by investigating the results of a resident-led DVT screening programme implemented at our ICU in April 2020. The secondary aim was to compare organ dysfunction, inflammation and coagulation between critically ill COVID-19 patients with and without venous thromboembolism (VTE).

## Material and methods

### Study design and patient population

This was an observational study of patients admitted to a mixed medical and surgical ICU who were screened for DVT in a clinically implemented programme between 10th April and 14th July 2020 at Uppsala University Hospital, a tertiary care centre in Sweden. The study was performed as a sub-analysis of patients included in a cohort study (PronMed) [28] approved by the National Ethical Review Agency (EPM; 2020–01623). Written informed consent was obtained from patients, or next of kin if the patient was unable to give consent. The study was registered a priori 20 March 2020 (ClinicalTrials ID: NCT04316884).

All patients admitted to the ICU with COVID-19 diagnosis confirmed by positive SARS-CoV-2 reverse transcription polymerase chain reaction tests on naso- or oropharyngeal swabs were eligible for lower extremity DVT screening with proximal compression ultrasound (2-CUS or ECUS) as soon as possible after ICU admission and included in the present analysis if they were ≥ 18 years old and had respiratory failure (PaO_2_/FiO_2_ ratio ≤ 40 kPa (300 mmHg)) [29].

### Study setting

**Fig. 1:**
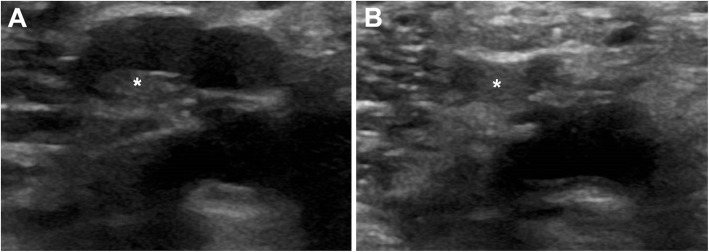
Screening ultrasound. The images display the bifurcation of the popliteal vein into the calf veins. The thrombus, marked with an asterisk (*), can be distinguished even without compression in the left vein (**a**). The left vein cannot be fully compressed, which is diagnostic of thrombosis, whereas the right vein demonstrates normal compression with full collapsibility (**b**). This pathological screening ultrasound was later confirmed by formal ultrasound

ICU residents were recruited to a DVT screening programme and received a 25-min online video tutorial followed by a single hands-on session supervised by a physician (JR) certified in echocardiography but with limited experience in DVT studies (20 examinations). 2-CUS and ECUS were taught according to consensus guidelines [10] and performed using high frequency linear array probes on GE Logiq S8 (GE Healthcare, Chicago, IL, USA) or Philips Sparq (Philips Ultrasound. Inc., Bothell, WA, USA) ultrasound machines. The ultrasound examination was considered pathological if compression did not cause complete collapse of the examined vein (Fig. 1). Patients with pathological screening were referred for formal CDUS and patients with negative screening were followed up and referred for formal ultrasound on clinical suspicion.

Critically ill COVID-19 patients in our ICU received weight-based low molecular weight heparin (LMWH) thromboprophylaxis with dalteparin sodium (Fragmin, Pfizer, New York, NY, USA). Patients < 50 kg received 5000 international units (IU) daily, patients 50–90 kg received 10,000 IU daily and patients > 90 kg received 150 IU/kg daily. Alterations in LMWH dosage was made according to anti-factor Xa-activity assays every three to seven days (COVID-19 modified target ranges per local guidelines: 0.50–0.80 kIE/L for prophylaxis (normally 0.20–0.45 kIE/L) and 0.90–1.20 kIE/L for treatment of confirmed VTE (normally 0.60–0.90 kIE/L)). Anti-factor Xa-assays were sampled three hours after LMWH dosing and were run using STA®-Liquid Anti-Xa (Stago, Asnières-sur-Seine Cedex, France) [30]. D-dimer values were not used to modify LMWH dosage. The D-dimer reagent used was STA® -Liatest® D-Di Plus (Stago, Asnières-sur-Seine Cedex, France) [31].

### Data collection

Information regarding demographic data, medical history, comorbidities, anticoagulation, DVT screening, subsequent DVT diagnosis, computed tomography angiography (CTPA) results and mortality was collected from electronic medical records until 90 days after inclusion, transfer to another hospital or time of death. Laboratory data were extracted from medical records during ICU stay.

### Statistical methods

Statistical analyses were performed in Microsoft Excel (Redmond, WA, USA) and R, version 3.6.3 (R Commander, R Foundation for Statistical Computing, Vienna, Austria, https://www.R-project.org/). Continuous variables were expressed as means and standard deviations (SD) or medians and interquartile ranges (IQR) as appropriate. Categorical variables were expressed as numbers and percentages. Normally and non-normally distributed continuous data was compared using independent t-test and Mann-Whitney U-test respectively and Fisher’s exact tests were used to compare categorical data. Two-sided *p*-values < 0.05 were considered statistically significant.

## Results

### Patient characteristics

Of 90 eligible patients, 56 were screened for DVT between the 27th of April and the 14th of July 2020. Mean age was 62.3 (SD 13.5) years, median body mass index was 29.0 (IQR 26.7–34.0) kg/m^2^ and 13 (23%) patients were female (Table 1). The most common comorbidities were hypertension, pulmonary disease and diabetes mellitus. Two patients (3.6%) had a medical history of combined DVT and PE prior to COVID-19.The median duration of symptoms before ICU admission was 10 days (IQR 8–12) and the median Simplified Acute Physiology Score (SAPS 3) [32] on admission was 53 (SD 10). Forty-six patients (82%) received thromboprophylaxis with subcutaneous injections of dalteparin and five patients (8.9%) received therapeutic anticoagulation with dalteparin or unfractionated heparin prior to screening. Five patients (8.9%) did not receive anticoagulation prior to screening, either due to contraindication or because they were screened prior to administration of the first dose of LMWH.

**Table 1:** Patient characteristics for all patients and for patients diagnosed with VTE and those with no VTE during the ICU stay

Variable	Total (***n*** = 56)	No VTE (***n*** = 48)	VTE (DVT ± PE; ***n*** = 3, PE ***n*** = 5)
Age, years (SD)	62.3 (13.5)	61.6 (14.4)	65.5 (3.6)
Female sex n(%)	13 (23)	13 (27)	0 (0)
BMI, kg/m^2^ (IQR)	29.0 (26.7–34.0)	29.2 (26.6–34.1)	28.5 (27.2–31.6)
Hypertension n(%)	29 (52)	23 (48)	6 (75)
Pulmonary disease			
Asthma n(%)	10 (18)	9 (19)	1 (13)
COPD n(%)	5 (8.9)	5 (10)	0 (0)
Unspecified n(%)	1 (1.8)	1 (2.1)	0 (0)
Ischemic heart disease n(%)	7 (13)	6 (13)	1 (13)
History of VTE n(%)	2 (3.6)	1 (2.1)	1 (13)
Diabetes mellitus n(%)	12 (21)	10 (21)	2 (25)
Ongoing smoking n(%)	4 (7.3)	4 (8.5)	0 (0)
Previous smoking n(%)	12 (22)	10 (21)	2 (25)
Heart failure n(%)	2 (3.6)	2 (4.2)	0 (0)
Days from symptom onset at ICU admission, (IQR)	10 (8–12)	9 (8–11)	10 (9–13)
SAPS 3 score at ICU admission (SD)	53 (10)	53 (10)	52 (8)
Parenteral anticoagulation prior to DVT screening			
Prophylactic dose n(%)	46 (82)	43 (90)	3 (38)
Therapeutic dose n(%)	5 (8.9)	0 (0)	5 (63)

Data are presented as mean (standard deviation), median (interquartile range) and absolute numbers (percentages). *VTE* Venous thromboembolism, *BMI* Body mass index, *COPD* Chronic obstructive pulmonary disease, *PE* Pulmonary embolism, *DVT* Deep vein thrombosis, *ICU* Intensive care unit, SAPS 3: Simplified Acute Physiology Score 3 [32]

### Ultrasound screening

Seven ICU residents volunteered to participate in the screening programme. Two physicians had limited experience (10–20 prior examinations) of DVT ultrasound, whereas the other physicians had none. They performed a median of 4 (IQR 2–19) screening ultrasounds. Patients were screened with either 2-CUS (61%) or ECUS (39%) at median day 3 (IQR 1–5) after ICU admission. Four patients (7.1%) had a pathological screening result, out of which three (5.4%) were corroborated by formal CDUS. Two residents with no previous DVT ultrasound experience had one true positive screening examination each. One resident with limited prior experience had one true positive and one false positive examination. All confirmed DVT were localized in the popliteal veins and all but one was unilateral. None of the 52 patients with initial negative screening ultrasound were diagnosed with DVT during follow-up.

### Venous thromboembolism, inflammation and organ dysfunction

Thirty patients (54%) underwent at least one CTPA, of which thirteen (23%) had CTPA performed after ultrasound screening. Six patients were diagnosed with PE; four after negative screening, and two after negative and positive screening respectively.

Patients with VTE (DVT ± PE; *n* = 3, PE *n* = 5) were older, had higher peak plasma values of C-reactive protein (CRP), D-dimer and creatinine but no statistically significant difference in peak plasma values of troponin I and N-terminal pro brain natriuretic peptide (NT-pro-BNP) compared to patients without VTE (*n* = 48) (Table 2).

**Table 2:** Peak plasma values from laboratory data during ICU stay and comparison between patients with and without VTE

Laboratory data	Total (*n* = 56)	No VTE (*n* = 48)	VTE (DVT ± PE; *n* = 3, PE *n* = 5)	*p*-value
D-dimer, mg/L (IQR)	3.3 (1.7–8.1)	2.8 (1.7–7.2)	24.0 (14.2–50.5)	0.004
CRP, mg/L (SD)	296 (108)	285 (108)	363 (80)	0.033
Ferritin, μg/L (IQR)	2879 (1643–4617)	2781 (1643–4421)	3916 (2369–7537)	0.262
IL-6, ng/L (IQR)	207 (100–334)	193 (91–274)	302 (197–639)	0.096
Creatinine, μmol/L (IQR)	95 (78–146)	94 (78–131)	288 (131–328)	0.009
Troponin I, ng/L (IQR)	21 [10–93]	21 (10–76)	79 (20–172)	0.167
NT-pro-BNP, ng/L (IQR)	1135 (424–3603)	937 (406–3203)	3700 (1003–5630)	0.167

*Data are presented as mean (standard deviation) or median (interquartile range). VTE: Venous thromboembolism, DVT: Deep vein thrombosis, CRP: C-reactive protein, IL-6: Interleukin 6, NT-pro-BNP: N-Terminal pro brain natriuretic peptide. Laboratory reference ranges: CRP < 5 mg/L; D-dimer < 0,50 mg/L (non-age-adjusted); Ferritin male patients 25–310 μg/L, female patients 10–155* μg*/L (non-age-adjusted); IL-6 < 7,0 ng/L; plasma creatinine male patients 60–105* μmol*/L, female patients 45–90 μmol/L; NT-pro-BNP male patients < 230 ng/L, female patients < 330 ng/L (non-age-adjusted); Troponin I male patients < 35 ng/L, female patients < 16 ng/L*

More patients with VTE received continuous renal replacement therapy (CRRT) during their ICU stay than patients without VTE (*p* = 0.005) but there was otherwise no difference in ICU length-of-stay or the proportion of patients receiving mechanical ventilation or vasoactive treatment.

Eleven patients (20%) died during the ICU stay, and an additional three patients (5.5%) died within 90 days from inclusion. There were no differences in mortality for patients diagnosed with VTE compared to patients without VTE at ICU discharge or at three-month follow-up (Table 3). Seven patients (16%) were transferred to other hospitals, resulting in loss to follow-up for subsequent VTE diagnosis for all patients and vital status for one patient.

**Table 3:** Patient outcomes and comparison between patients with and without VTE

Outcomes	Total (*n* = 56)	No VTE (*n* = 48)	VTE (DVT ± PE; *n* = 3, PE ***n*** = 5)	*p*-value
ICU length-of-stay, days (IQR)	12 (6–20)	10 (6–18)	17 (15–25)	0.087
Died during ICU stay n(%)	11 (20)	9 (19)	2 (25)	0.649
Died within 90 days n(%)	14 (25)	12 (26)	2 (25)	> 0.999
Lowest PaO_2_/FiO_2_-ratio, kPa (IQR)	9.8 (8.6–11.0)	9.8 (8.2–11.0)	9.8 (9.4–11.1)	0.510
Vasoactive treatment n(%)	41 (73)	34 (71)	7 (88)	0.428
CRRT n(%)	11 (20)	6 (13)	5 (63)	0.005
Mechanical ventilation n(%)	35 (63)	28 (58)	7 (88)	0.236

Data are presented as median (interquartile range) or absolute numbers (percentages). *VTE* Venous thromboembolism, *PE* Pulmonary embolism, *DVT* Deep vein thrombosis, *ICU* Intensive care unit, *CRRT* Continuous renal replacement therapy

## Discussion

The main finding of this study was that ICU residents provided with a short education could find DVT in critically ill COVID-19 patients. This is, to our knowledge, the first study of DVT screening by physicians with no or limited previous experience in DVT ultrasound in this setting. Three of four positive findings were corroborated by formal ultrasound and no DVT were diagnosed in patients with negative screening ultrasound during follow-up, suggesting acceptable sensitivity, specificity and predictive values, whereas the small study sample and few events prohibits the calculation of precise estimates.

Two meta-analyses in non-COVID-19 settings have found that emergency physician-performed ultrasound had a high agreement with formal ultrasound and a sensitivity of 95–96% and specificity of 96–97% [12, 13]. An intensivist-performed ultrasound screening study for proximal DVT in trauma patients reported a positive predictive value of 92%, a negative predictive value of 97% and a specificity of 99% compared to formal ultrasound, corroborating our results [33]. However, the sensitivity was only 69% due to the occurrence of non-occlusive DVT, which are more difficult to detect because the veins will partially collapse with compression. Patients with negative screening were not referred for CDUS in our study, and small non-occlusive thromboses could thus have been missed.

We found DVT in 5.4% of screened patients. Previous screening studies in critically ill COVID-19 patients have found proximal lower extremity DVT in 10–23% of included patients [15, 20, 22, 26]. However, standard prophylactic dose of LMWH was used in these studies and repeated scanning in three studies led to additional DVT diagnoses [15, 20, 22], whereas in the present study, a higher-dose thromboprophylaxis regimen was used and screening was only performed once, which may at least in part explain our relatively low rate of diagnosed DVT.

Five patients were diagnosed with PE prior to or following negative screening ultrasound. This may have several explanations, including false negative screening, subsequent proximal leg thrombosis or emboli from other venous territories. Extensive ultrasound screening of all extremities and the central venous system will diagnose additional DVT compared to proximal lower extremity screening alone [24], although feasibility is limited in absence of experienced sonographers, and patients with PE may still be DVT negative [34] due to in-situ pulmonary immunothrombosis [35].

We found a higher peak D-dimer value in COVID-19 patients with VTE compared to patients who did not have VTE, in line with previous reports [4, 5, 22, 24]. Elevated D-dimer correlates with poor prognosis, and among patients with D-dimer> 3 mg/L the use of thromboprophylaxis is associated with lower mortality [2]. Patients diagnosed with VTE had higher peak CRP values compared to those who were not, consistent with a recent study [36]. We found no difference in interleukin-6 (IL-6) values between VTE and non-VTE patients, but the analysis may be hampered by low statistical power. However, LMWH lowers IL-6 levels [37] and the high LMWH doses used in this cohort could possibly influence these results.

Although patients with PE may present with right ventricular strain and elevated levels of cardiac biomarkers [38], we found no difference in peak values of troponin-I and NT-pro-BNP in patients with and without VTE in our study. COVID-19 is associated with other causes of myocardial injury than PE, including ischemia, hypoxemia, pulmonary hypertension and myocarditis, which are likely to attenuate differences in troponin-I and NT-pro-BNP values between VTE and non-VTE patients [39]. Also, patients with COVID-19 and PE are reported to have less clot burden and associated right ventricular strain compared to other patients with PE [35], which further may contribute to these findings.

Renal dysfunction and CRRT was more common in patients with VTE compared to patients without VTE. Both VTE [5, 23] and acute kidney injury (AKI) [40] are more common in severely ill COVID-19 patients compared to patients with mild disease. AKI is further associated with higher levels of biomarkers of inflammation and coagulation and in-hospital death in COVID-19 patients [41, 42]. AKI and VTE may thus both reflect severity of disease, or one may contribute to the development of the other. We found no difference in mortality or other supportive treatments (mechanical ventilation, vasoactive treatment) between patients with and without VTE, but our study is likely underpowered to detect such differences.

Strengths of our study include that all physicians in the screening programme were residents with very limited experience of DVT ultrasound that were given a brief ultrasound education. In our experience, most ICU physicians are not proficient in DVT-ultrasound. This study therefore probably reflects the pre-existing level of experience and screening implementation process at most centres during the COVID-19 pandemic, which increases generalizability.

Our study also has limitations. The small sample size decreases statistical power and the single centre setting reduces generalizability. Not all eligible patients were included, which may have led to selection bias, and undiagnosed fatal PE may have led to underestimation of the incidence of VTE [43]. Using DVT diagnosed during follow-up instead of formal ultrasound in cases of negative screening may have led to missed false negatives. One positive screening was not confirmed on formal ultrasound. This underscores the need for confirmation of pathological screening ultrasound and that negative screening should not defer formal examination when there is clinical suspicion of DVT. However, all DVT cases discovered in this study would likely have been missed and not received adequate treatment without screening, indicating possible benefit for patients. The present study may serve as a basis for future larger studies which may define estimates for sensitivity, specificity and predictive values.

## Conclusion

ICU residents with limited experience in DVT ultrasound could detect DVT in critically ill COVID-19 patients following a brief education session. VTE was associated with more severe kidney dysfunction, more marked inflammatory response and features of hypercoagulation. Point-of-care ultrasound screening for DVT may be a resource-sparing alternative to expert CDUS screening in the setting of the COVID-19 pandemic.

## Data Availability

The datasets used and/or analyzed during the current study are available from the corresponding author on reasonable request (10.17044/scilifelab.14229410).

## Abbreviations

AKI: Acute kidney injury
BMI: Body mass index
CDUS: Complete duplex ultrasound
CRP: C-reactive peptide
CRRT: Continuous renal replacement therapy
CTPA: Computed tomography pulmonary angiography
2-CUS: Two-region compression ultrasound
DVT: Deep vein thrombosis
ECUS: Extended compression ultrasound
ICU: Intensive care Unit
IL-6: Interleukin 6
IU: International unit
IQR: Interquartile range
LMWH: Low molecular weight heparin
NT-pro-BNP: N-Terminal pro brain natriuretic peptide
PE: Pulmonary embolism
SD: Standard deviation
VTE: Venous thromboembolism
